# Does Meditation Alter Brain Responses to Negative Stimuli? A Systematic Review

**DOI:** 10.3389/fnhum.2018.00448

**Published:** 2018-11-13

**Authors:** Andressa A. Magalhaes, Leticia Oliveira, Mirtes G. Pereira, Carolina B. Menezes

**Affiliations:** ^1^Laboratório de Neurofisiologia do Comportamento, Instituto Biomédico, Universidade Federal Fluminense, Niterói, Brazil; ^2^Departamento de Psicologia, Centro de Filosofia e Ciências Humanas, Universidade Federal de Santa Catarina, Florianópolis, Brazil

**Keywords:** emotion regulation, emotional reactivity, fMRI, sitting and silent meditation, aversive stimuli, mindfulness

## Abstract

**Background:** Despite several attempts to review and explain how meditation alters the brain and facilitates emotion regulation, the extent to which meditation and emotion regulation strategies share the same neural mechanisms remains unclear.

**Objective:** We aim to understand the influence of meditation on the neural processing of negative emotional stimuli in participants who underwent meditation interventions (naive meditators) and long-term meditators.

**Methodology:** A systematic review was conducted using standardized search operators that included the presence of terms related to emotion, meditation and neuro-imaging techniques in PsycInfo, PubMed, Scopus, and Web of Science databases.

**Results:** Searches identified 882 papers, of which 11 were eligible for inclusion. Results showed a predominance of greater prefrontal/frontal activity related to meditation, which might indicate the increased recruitment of cognitive/attentional control resources in naïve and long-term meditators. This increased frontal activity was also observed when participants were asked to simply react to negative stimuli. Findings from emotion-related areas were scarce but suggested increased insular activity in meditators, potentially indicating that meditation might be associated with greater bodily awareness.

**Conclusions:** Meditation practice prompts regulatory mechanisms when participants face aversive stimuli, even without an explicit request. Moreover, some studies reported increased insular activity in meditators, consistent with the hypothesis that meditation helps foster an interoceptive awareness of bodily and emotional states.

## Introduction

Meditation practices have become a popular and widely investigated psychotherapeutic technique and form of general health promotion. Motivated by traditional and philosophical claims that meditation helps people relieve suffering and achieve well-being (Wallace and Shapiro, 2006), these practices have been adapted and incorporated into programs that aim to foster healthy psychological functioning and help people overcome emotional problems (Chiesa and Malinowski, 2011).

From both traditional and scientific perspectives, there is a well-established diversity in meditation practices, which can vary according to the mental procedure they use (e.g., orientation, visualization, recitation, focusing on body movement, generating feelings), the way these procedures are used (e.g., effortlessly, actively, internally, guided), and the phenomena to which the mental activity is directed (e.g., thoughts, images, concepts, part of the body, sensation, deity) (Lutz et al., 2015; Guendelman et al., 2017). In addition to these variances, there has been great debate concerning the cognitive mechanisms underlying these practices. Frequently mentioned distinctions refer to the type of mental skill employed during the actual practice, such as how attention is cultivated (concentration vs. awareness) and which or whether “appraisal processes” occur (non-reflexive activity in which one only observes cognitions from a detached perspective vs. deliberately modifying cognitions) (Lutz et al., 2007).

Within a scientific framework, meditation practices are generally grouped into two primary types, described as focused attention (FA) and open monitoring (OM) meditation (Slagter et al., 2011). FA practices aim to develop mental stability by systematically training selective attention (i.e., focusing on one thing at once) and sustained attention (i.e., paying attention over extended periods of time). During FA, meditators attend to a specific object such as the breath or a symbol and return attention to the selected object every time they notice their mental activity has wandered. OM practices characterize metacognitive processes in which one is trained to non-judgmentally monitor cognitive and emotional events, thus cultivating a detached awareness of experiences. More recently, some authors have attempted to outline a more complex typology of meditation practices to better understand the distinct cognitive processes underlying each style and how these cognitive processes impact emotion regulation (Dahl et al., 2015). According to this model, there are three categories of meditation practices. FA (e.g., breath counting or Shamatha) and OM (e.g., components of mindfulness-based programs) practices are grouped into an attentional category, which involves processes such as attention regulation and meta-awareness. The other two categories are named constructive and deconstructive and refer respectively to the processes of perspective taking/reappraisal (e.g., loving-kindness and compassion meditation) and self-inquiry (e.g., analytical meditation or Koan practice). These authors argue that although many practices contain elements of all three categories, these classifications are based on the primary mechanisms each style employs during actual practice.

Undoubtedly, the most widely used and investigated practices in the clinical context are FA and OM (Davidson and Kaszniak, 2015) from the attentional category; more recently, there has been increased interest in loving-kindness practices (Hofmann et al., 2011) from the constructive category. Despite sharing common goals, such as promoting well-being, empirical evidence supports the notion that these categories may indeed affect distinct cognitive processes (Feldman et al., 2010). Considering the clinical relevance of attentional practices (Guendelman et al., 2017) and the need to advance the understanding of the neural correlates of the attentional category (Davidson and Kaszniak, 2015), the present work will focus on FA and OM meditation styles.

Several researchers have attempted to review and explain how meditation alters the brain (Hölzel et al., 2011; Vago and Silbersweig, 2012; Fox et al., 2014, 2016; Tang et al., 2015) and how it facilitates emotion regulation (Chiesa et al., 2013; Grecucci et al., 2015; Gu et al., 2015; Guendelman et al., 2017). Overall, these studies suggest that practices from the attentional category induce changes in brain structure and function, particularly in the dorsal anterior cingulate cortex (which is possibly related to general processes of self-regulation, increased attention to thoughts and information entering decision-making process and to the body during action execution), insula (linked to an enhancement of body awareness), dorsolateral prefrontal cortex (associated with introspection and enhancement in metacognitive skills), and the default mode network. The default mode network is the most active brain system when subjects are allowed to believe that they are undisturbed and when the demands to process environmental information are very low. The posterior cingulate cortex, the ventromedial and dorsomedial prefrontal cortex, the inferior parietal lobule, the lateral temporal cortex and hippocampus are the core brain regions of this default network (Raichle et al., 2001; Buckner et al., 2008). Meditation is associated with structural differences and reduced activity of the default mode network and might reflect less mind-wandering and reduced chaining of thoughts in long-term meditators (Brewer et al., 2011; Kang et al., 2013). Overall, theoretical models derived from these findings have proposed that self-regulation is a key process by which meditation fosters improved emotional functioning (Hölzel et al., 2011; Vago and Silbersweig, 2012; Tang et al., 2015).

Regarding the neural substrates underlying the perception of emotional stimuli, a large body of studies has shown increased activation of the amygdala (Ochsner et al., 2002; Phan et al., 2002; Hariri et al., 2003; Phelps and LeDoux, 2005; Eippert et al., 2007; Schirmer and Adolphs, 2017). This region has consistently been implicated in the emotional processing of negative and positive stimuli and the facilitation of attentional orientation to the emotional relevance of these stimuli (Vuilleumier, 2005; Phelps, 2006; Pessoa, 2008). The insula is another brain area that seems important to the identification of emotional significance, and its increased activity has been described in the anticipation of aversive stimuli and when subjects encounter a threatening object (Phelps et al., 2001; Mobbs et al., 2010; Holtz et al., 2012; Liljeholm et al., 2014; Sánchez-Álvarez et al., 2015). Therefore, threat and the risk of threat are both potential factors that engage the insula (McNaughton and Corr, 2004). Furthermore, increased activation of the insula observed in emotional contexts is associated with the monitoring of the ongoing internal emotional states (Craig, 2009). In addition to the amygdala and insula, other structures, such as the ventral anterior cingulate cortex and ventromedial prefrontal cortex, have also been suggested as being involved in emotional processing and/or the generation of affective responses (Phan et al., 2002; Phillips et al., 2003).

Emotion regulation strategies, such as cognitive reappraisal, implicit cognitive reappraisal, and attentional deployment, have been shown to promote more adaptive responses to emotional content (Gross, 1998; Mauss et al., 2007). Investigations of neural activity during the use of these strategies have revealed patterns of increased prefrontal activity and/or decreased activity of the limbic region (Mocaiber et al., 2011; Ferri et al., 2013, 2016; Wang et al., 2017). For example, a meta-analysis of neuroimaging studies revealed that the use of the cognitive reappraisal strategy consistently activated regions involved in cognitive control (dmPFC, d1PFC, vlPFC, and posterior parietal lobe) and the lateral temporal cortex, and modulated activity in the amygdala (Buhle et al., 2014). Accordingly, Ferri et al. (2013) observed increased activity in fronto-parietal regions and reduced activity in the amygdala when participants were instructed to change their emotional responses through attentional deployment. Another strategy, called implicit reappraisal, also showed this pattern of neural activity when participants faced negative stimuli (Mocaiber et al., 2011; Wang et al., 2017).

The present review is innovative as we focused on how the formal practice of sitting and silent meditation influences the neural processing of negative emotions, evoked by visual stimuli presentation. Previous reviews exploring how neural mechanisms underlying meditation and emotion regulation are related have grouped a great variety of meditation practice styles and experimental designs, such as different type of emotional stimuli and/or evoked by different sensory modalities. Moreover, to our knowledge, this is the first review that explores if the neural networks modulated by meditation when participants were instructed to regulate their emotional response are different from those modulated when participants naturally react to the aversive stimuli.

## Methods

### Eligibility criteria

No constraints regarding population were implemented; thus, participants of all ages and those who were both healthy or with a clinical condition were included in the review. Our aim was to review studies that only examined meditation practice styles that fell into the attentional category. Therefore, commonly investigated practices, such as compassion meditation, were not included. In addition, mindfulness-based interventions (MBIs) combined with psychotherapeutic techniques (e.g., mindfulness-based cognitive therapy, mindfulness-based relapse prevention, and mindfulness-oriented recovery enhancement) were excluded, because therapeutic procedures aim to directly influence emotion regulation skills, making the isolation of the regulatory effects of meditation techniques difficult. In summary, this review only considered studies that assessed the following parameters to avoid high levels of heterogeneity: (1) FA and OM practices (attentional category); (2) the effects of the actual practice, excluding investigations of dispositional mindfulness; and (3) brain reactivity to negative visual stimuli.

We considered studies that investigated naïve participants who underwent a meditation intervention (i.e., experimental designs) as well as long-term practitioners (i.e., quasi-experimental designs). For experimental designs, there were no limits on the length of the interventions, and participants could not have had experience with meditation prior to the intervention. For the quasi-experimental studies, meditators should have had a minimum lifetime practice amount (more than 1,000 h or 1 year of practice). For all studies, practitioners had to be compared with at least one control group.

The present systematic review examined the effect of meditation practice on neural activity during the processing of emotional stimuli. To reach greater homogeneity for better comparisons between studies and taking into account the clinical impact that negative emotions may have on mental health, only studies that used tasks with negative visual stimuli were included. To investigate the neural correlates of emotional processing, only studies using neuroimaging techniques, such as diffusion tensor imaging, voxel-based morphometry, functional magnetic resonance imaging, and positron emission tomography, were included. Connectivity and temporal dynamic analyses were not included.

### Search strategy

A comprehensive search of studies published up to January 2018 was conducted using the following electronic databases: PubMed, PsycInfo, Scopus, and Web of Knowledge. The search terms were (meditat^*^ OR mindful^*^ OR yoga^*^) AND (emot^*^ OR affect^*^) AND (neuroimaging OR “diffusion tensor image” OR DTI OR “voxel-based morphometry” OR VBM OR “functional magnetic resonance” OR fmri OR “positron emission tomography” OR PET). Only reports published in peer-reviewed scientific journals were included (results from conference abstracts, presented talks, dissertations, etc., were excluded), and those that reanalyzed data that had already been included were not considered for this review. We did not impose any limits on the date of publication.

### Study selection and data extraction

Search terms were entered into the databases, and duplicate studies were removed. The titles of the remaining studies were read, and those outside of our focus (different design, different themes, etc.) were excluded. The abstracts were independently assessed by two reviewers who verified if they met the inclusion criteria. Disagreements were resolved by consensus or, in the absence of consensus, by the decision of a third reviewer. The two reviewers then read the full articles that were potentially relevant for the systematic review, and in cases of persisting disagreements, the third reviewer was consulted.

Results related to the modulation of neural activity during the processing of negative emotional stimuli were extracted, taking into account the regions that were altered as well as the direction in which the activity occurred in these regions (increases or decreases).

To better understand the effects of meditation on neural responses, findings were organized according to study design (experimental and quasi-experimental). Additionally, studies using emotional paradigms can generate different types of outcomes: some assess the passive viewing of stimuli (reactivity), others explicitly require participants to regulate the processing of emotional stimuli (regulation), and some assess both conditions (reactivity and regulation). Thus, for each type of design, the results were also organized according to type of outcome of the emotional paradigm, namely, emotional reactivity or emotion regulation.

## Results

**Figure 1:**
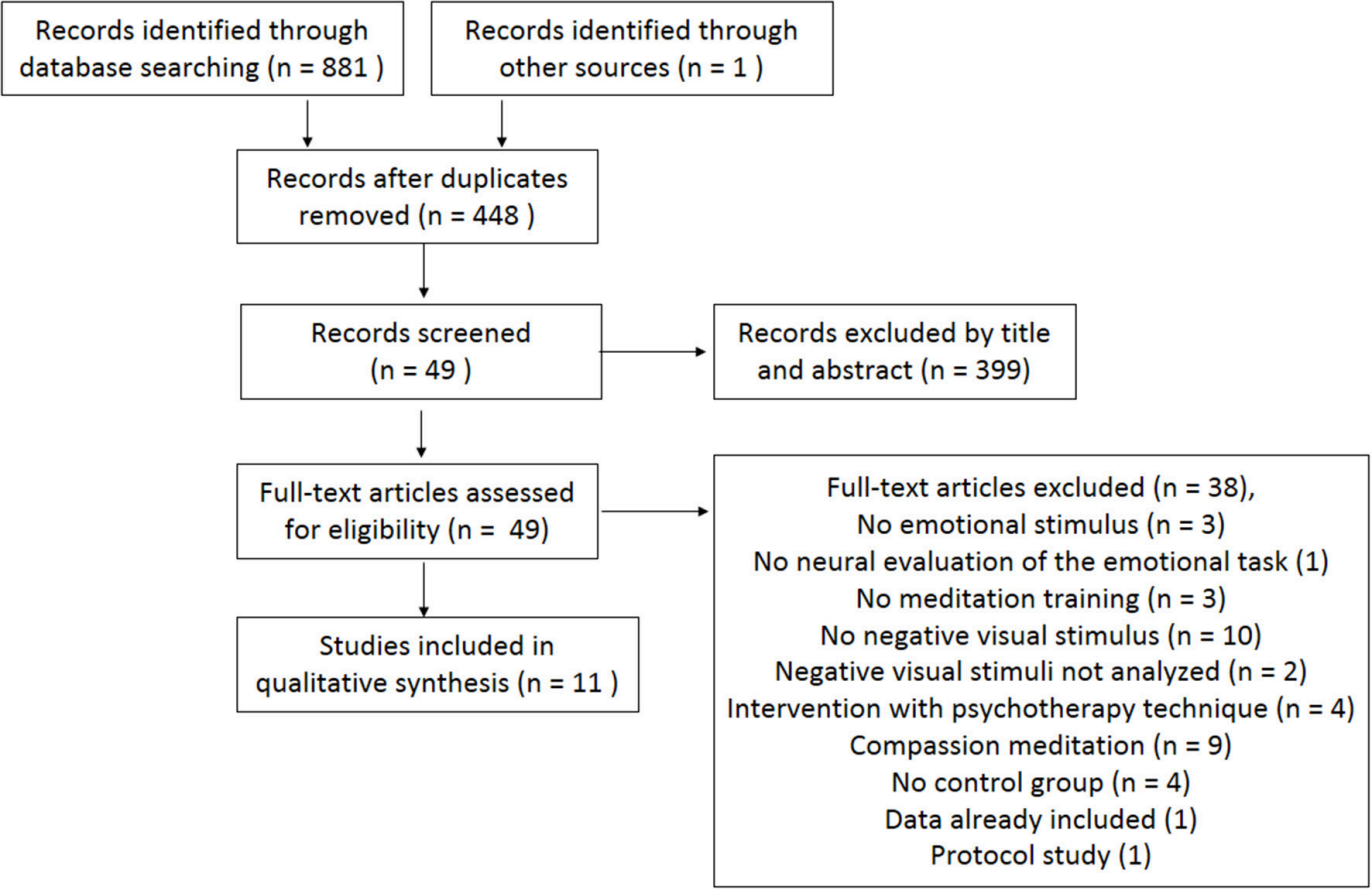
PRISMA flow diagram of the literature search (Moher et al., 2009).

The initial search generated 881 studies, and one study was retrieved from the reference list of one of those papers. After removing duplicates, 448 articles remained. After reading titles and abstracts, 399 articles were excluded. For the 49 potentially relevant articles, two reviewers read the whole paper and excluded 38 for the following reasons: did not include emotional stimuli (3); did not assess neural responses during exposure to emotional stimuli (1); did not assess the actual meditation practice (3); did not use visual negative emotional stimuli (10); did not analyze visual negative stimuli (2); used interventions that were complemented by psychotherapeutic techniques (4); assessed compassion meditation (9); did not have a control group (4); comprised data already included (1); and comprised a protocol study (1). Hence, 11 articles were eligible and included in the current systematic review (see the flow diagram in Figure 1).

### Characteristics of the included studies

#### Study design

Of the 11 included studies, six used experimental design, that is, assessed the effect of a meditation intervention in comparison with a control group. Five of these studies assessed participants before and after the intervention (Allen et al., 2012; Desbordes et al., 2012; Goldin et al., 2012, 2013; Hölzel et al., 2013), and one collected data only after the intervention (Farb et al., 2010). The other five studies relied on a quasi-experimental design, in which there was no random distribution of participants and no intervention manipulation because participants were already long-term meditators (Taylor et al., 2011; Froeliger et al., 2012; Lee et al., 2012; Lutz et al., 2016; Laneri et al., 2017).

#### Samples

Three articles investigated participants who presented some clinical condition. Two of these included participants with social anxiety disorder according to the diagnostic criteria of the DSM IV (Goldin et al., 2012, 2013), and the third study (Hölzel et al., 2013) assessed participants with generalized anxiety disorder as indexed by the clinical interview of the DSM IV (*Structured Clinical Interview for DSM IV*—SCID). The remaining studies included healthy samples (Taylor et al., 2011; Allen et al., 2012; Desbordes et al., 2012; Froeliger et al., 2012; Lee et al., 2012; Lutz et al., 2016; Laneri et al., 2017) except for one study, which did not provide the samples' health status (Farb et al., 2010).

#### Control groups

Five of the six experimental studies included an active control group whose activity varied by study: reading (Allen et al., 2012), discussions about health issues (Desbordes et al., 2012), education on stress management (Hölzel et al., 2013), and aerobic exercise (Goldin et al., 2012, 2013). The study by Farb et al. (2010) used a waitlist control group.

Among the five quasi-experiments, three compared the long-term meditators with a non-meditators control group (Froeliger et al., 2012; Lutz et al., 2016; Laneri et al., 2017). The other two instructed participants from the control group to practice meditation at home to compare long-term practitioners with beginners. Of these studies, one required participants to meditate mindfulness for 20 min for 7 days (Taylor et al., 2011), and the other instructed participants to practice three 20-min sessions per day for 7 days (Lee et al., 2012).

#### Type of meditation practice and duration

Among the six experimental studies, four investigated the Mindfulness-Based Stress Reduction Program (MBSR) (Farb et al., 2010; Goldin et al., 2012, 2013; Hölzel et al., 2013), and the other two assessed mindfulness-based practices (Allen et al., 2012; Desbordes et al., 2012). Most of interventions lasted 8 weeks except one study that conducted a 6-week intervention (Allen et al., 2012). Interventions consisted of weekly meetings lasting from two to two and a half hours, and participants were always instructed to practice at home. Only one study did not require home practice (Farb et al., 2010).

The quasi-experimental studies assessed the following practices: Zen (Taylor et al., 2011), Vipassana (Lutz et al., 2016), Zen and Vipassana (Laneri et al., 2017), Hatha Yoga (Froeliger et al., 2012), and Theravada (Lee et al., 2012).

All practices from the studies included in the current review fell under the attentional category and consisted of either FA or OM meditations (Lutz et al., 2008).

#### Visual emotional stimuli

With respect to the visual stimuli used in the emotional paradigms, five studies used International Affective Picture System (IAPS) images (Taylor et al., 2011; Allen et al., 2012; Desbordes et al., 2012; Froeliger et al., 2012; Lee et al., 2012), one study used faces (Hölzel et al., 2013), one study used video with audio (Farb et al., 2010), three studies presented written adjectives (Goldin et al., 2012, 2013; Lutz et al., 2016), and one study used sketches accompanied by phrases (Laneri et al., 2017). See Table 1 for details of included studies.

**Table 1:** Characteristics of included studies.

**Study**	**Sample handedness reported**	**Sample gender reported**	**Sample (*n*) meditators/controls**	**Participants had history of psychiatric disorder**	**Current use of any pcychoative medications**	**Sampling strategy described**	**Instructor(s) features described**	**Participants payment described**
1	Y	Y	(19)/(19)	N	N	Y	Y	Y
2	N	Y	(12)/(12)	Unclear	Unclear	Y	N	N
3	Y	Y	(20)/(16)	Unclear	Unclear	Y	N	N
4	Y	Y	(15)/(11)	Y	Y	Y	N	N
5	N	Y	(24)/(18)	Y	N	Y	Y	N
6	N	Y	(23)/(19)	Y	N	Y	Y	N
7	Y	Y	(7)/(7)	N	Unclear	N	N/A	N
8	Y	Y	(20)/(21)	N	N	Y	N/A	Y
9	Y	Y	(12)/(8)	N	N	Y	N/A	Y
10	Y	Y	(11)/(11)	N	Unclear	Y	N/A	N
11	N	Y	(32)/(16)	N	N	Y	N/A	N

*(1: Allen et al., 2012; 2: Desbordes et al., 2012; 3: Farb et al., 2010; 4: Hölzel et al., 2013; 5: Goldin et al., 2012; 6: Goldin et al., 2013; 7: Froeliger et al., 2012; 8: Lutz et al., 2016; 9: Taylor et al., 2011; 10: Lee et al., 2012; 11: Laneri et al., 2017)*.

### Neural responses

**Figure 2:**
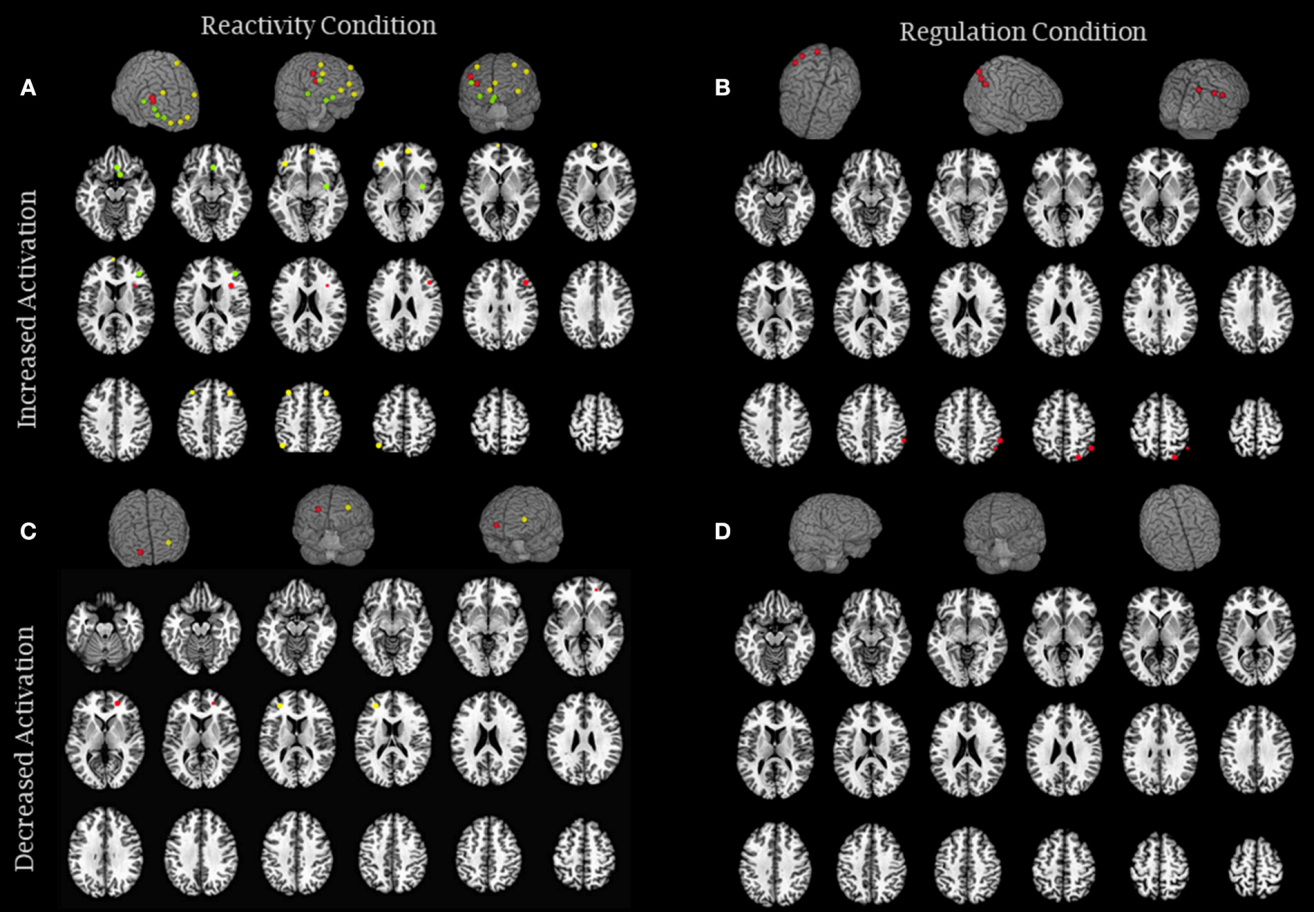
Brain activity modulation due to meditation intervention: experimental studies. **(A)** The image represents brain areas that showed increased activity in reactivity conditions (Farb et al., 2010—green; Goldin et al., 2012—yellow and Hölzel et al., 2013—red) and **(B)** increased activity in regulation conditions (Goldin et al., 2013—red). **(C)** The image represents brain areas that showed decreased activity in reactivity conditions (Farb et al., 2010— yellow and Goldin et al., 2013—red), and **(D)** decreased activity in regulation conditions (none). Each sphere was centered on the coordinates reported in the studies using a 5 mm radium. Areas are displayed on a rendered template brain provided by AFNI and on axial slices of the TTN27 AFNI template. Some results described in Table 2 had no coordinates reported in the original manuscripts precluding them to be included in this figure.

**Figure 3:**
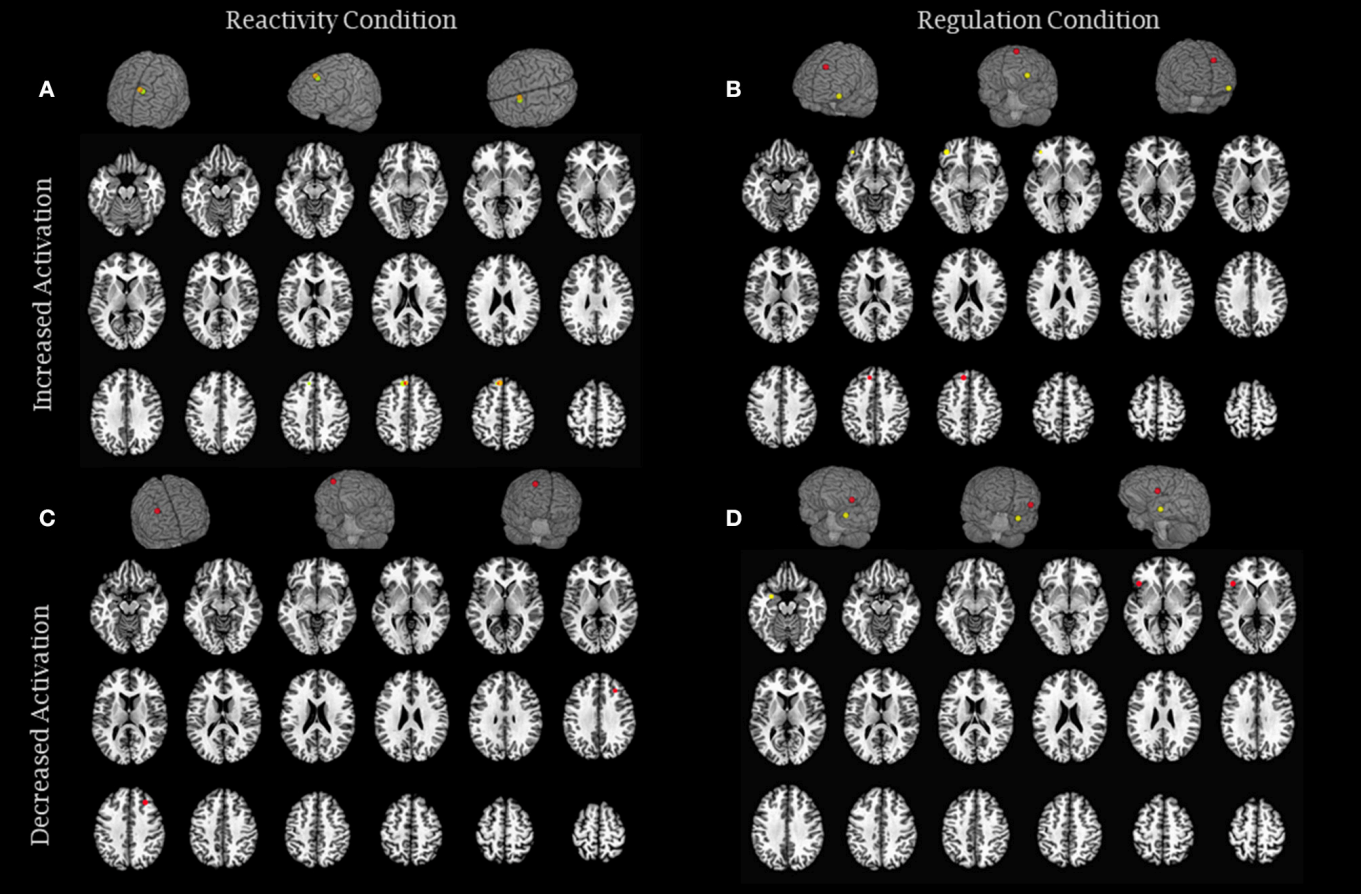
Brain activity modulation due to long-term meditation practice: quasi-experimental studies. **(A)** The image represents brain areas that showed increased activity in reactivity conditions (Lee et al., 2012—green and Lutz et al., 2016—orange) and **(B)** increased activity in regulation conditions (Froeliger et al., 2012—yellow and Lee et al., 2012—red). **(C)** The image represents brain areas that showed decreased activity in reactivity conditions (Froeliger et al., 2012—red), and **(D)** decreased activity in regulation conditions (Laneri et al., 2017—red and Taylor et al., 2011—yellow). Each sphere was centered on the coordinates reported in the studies using a 5 mm radium. Areas are displayed on a rendered template brain provided by AFNI and on axial slices of the TTN27 AFNI template. Some results described in Table 3 had no coordinates reported in the original manuscripts precluding them to be included in this figure.

All studies that met our inclusion criteria used functional magnetic resonance imaging to analyze neural responses. In the following sections, data are presented according to study design: *experimental studies* investigated naïve participants with no prior experience with meditation who underwent an intervention to learn the practice (see the experimental results in Table 2 and Figure 2), and *quasi-experimental studies* investigated long-term practitioners who had prior meditation experience (over 1,000 h or more than 1 year) (see the quasi-experimental results in Table 3 and Figure 3). Within each study design, data were also divided according to the type of emotional processing assessed during the paradigm: *emotional reactivity*, which consisted of the passive viewing of emotional stimuli, without any instruction to change or modulate the experience; and *emotion regulation*, in which participants were explicitly instructed to use some strategy to attempt to modulate the impact of the emotional stimuli. Considering that our aim was to understand the effects of meditation on the neural responses to negative emotions, the results reported are only those related to negative stimuli, even if the paradigm also used positive stimuli.

**Table 2:** Neural activity for either reactivity or regulation of negative emotional stimuli in experimental studies.

**Study**	**Meditation practice/Control**	**Daily practice**	**Stimuli**	**Comparison**	**Reactivity condition**	**Activity direction**	**Comparison**	**Regulation condition**	**Activity direction**
Allen et al., 2012	MT (6 weeks) Reading Group (6 weeks)	20 min/day	IAPS images	MT (post) x Reading Group (post)	^*^		–	
Desbordes et al., 2012	MT (8 weeks) Health discussion (8 weeks)	20 min/day	IAPS images	MT (post) x Health discussion (post)	^*^		–	
Farb et al., 2010	MBSR (8 weeks) Wait list	Instructed to practice at home	Film clips	MBSR (post) x Wait list	Right insula, the right subgenual ACC/gyrus rectus extending into the vmPFC, the right vlPFC, right SFG Left lateral PFC, frontal operculum/Broca's area, superior temporal Sulcus/Wernicke's area, inferior temporal gyrus	↑ ↓	–	
Hölzel et al., 2013	MBSR (8 weeks) SME (8 weeks)	Instructed to practice at home	Pictures	MBSR (post) x SME (post)	Right pars opercularis reaching insula, right rostral middle FC	↑	–	
Goldin et al., 2013	MBSR (8 weeks) Aerobic exercise (8 weeks)	Instructed to practice at home	adjectives	MBSR (post) x Aerobic Exercise (post)	Right vlPFC	↓	MBSR(post) x MBSR (pre)	Right posterior STG, bilateral lingual gyrus Left IPL, right anterior IPL, right posterior IPL, right superior PL	↓ ↑
Goldin et al., 2012	MBSR (8 weeks) Aerobic exercise (8 weeks)	Instructed to practice at home	adjectives	MBSR (post) x MBSR (pre)	PCC, vmPFC, lefl vlPFC, bilateral dlPFC, left IPL, left posterior STG	↑	–	

*ACC, anterior cingulate cortex; dlPFC, dorsolateral prefrontal cortex; FC, frontal cortex; IPL, inferior parietal lobule; MT, mindfulness training; PCC, posterior cingulate cortex; SFG, superior frontal gyrus; SME, stress management education; STG, superior temporal gyrus; vlPFC, ventrolateral prefrontal cortex; vmPFC, ventromedial prefrontal cortex; –, Did not investigate*;

* *, No results found*.

**Table 3:** Neural activity for either reactivity or regulation of negative emotional stimuli in quasi-experimental studies.

**Study**	**Meditation practice /Control**	**Daily practice of Beginners**	**Stimuli**	**Comparison**	**Reactivity condition**	**Activity direction**	**Comparison**	**Regulation condition**	**Activity direction**
Froeliger et al., 2012	Yoga (5.7 years) Control	n/a	IAPS images	Meditators x Controls	Right dlPFC	↓	Meditators x Controls	Left vlPFC	↑
Lutz et al., 2016	Mindfulness (mean: 4861.50 h) Control	n/a	adjectives	Meditators x Controls	dmPFC	↑	–	
Taylor et al., 2011	Zen (>1000 h) Beginners	20 min/day for a week	IAPS images	Meditators x Beginners	No difference between groups		Beginners (m) x Meditators (m)	Left amygdala	↓
Lee et al., 2012	Focused attention (>5 years) Beginners	1 h/day for a week	IAPS images	Meditators x Beginners	Left SFG	↑	Meditators (m) x Beginners (b)	Left SFG	↑
Laneri et al., 2017	Zen and Vipassana (5 to 38 years) Control	n/a	Hand-drawn Sketches	Meditators x controls	No difference between groups		Meditators (m) x Meditators (b)	Left AI	↓

*AI, anterior insula; b, baseline; dmPFC, dorsomedial prefrontal cortex; m, meditative state; n/a, not applicable; -, Did not investigate*.

#### Experimental studies

##### Emotional reactivity

The study by Allen et al. (2012) performed whole-brain analysis of BOLD (blood oxygenation level-dependent) signals to compare a mindfulness meditation intervention group with a reading control group. Participants performed an emotional Stroop task with neutral and negative images before and after the intervention. In the reactivity condition, in which participants only viewed the images, there were no significant interactions between group and time in the whole-brain analysis. However, there was a main effect for the meditation group, where the amount of practice (hours) predicted greater activity in the right anterior insula, medial prefrontal cortex (mPFC), and dorsal anterior cingulate cortex (dACC). Hence, despite no mean difference between groups, and despite no explicit instructions to regulate emotion, meditators with more hours of practice were better able to recruit frontoinsular regions, suggesting an enhanced capacity to modulate the processing of negative emotional stimuli.

Desbordes et al. (2012) investigated amygdala activity through ROI (region of interest) analysis. The experimental group underwent a mindfulness training, and the control group participated in meetings comprised of health discussions. Both groups viewed positive, negative and neutral images. When viewing negative images, no significant differences were found between groups in either left or right amygdala activity. The meditation group did show a reduction in amygdala activity in response to negative images compared to the control group, but this difference was not statistically significant.

Farb et al. (2010) compared a group who received the MBSR and a waitlist control group. All participants watched neutral and sad videos. In comparison to the control group, the meditation group showed increased activity in the ventromedial PFC (vmPFC), right ventrolateral PFC (vlPFC), right superior frontal gyrus (SFG), and the right basal ganglia extending to the right insula (mid to posterior insula) and right subgenual ACC and reduced activity in the right precuneus/posterior cingulate, and left-lateralized reductions in the prefrontal cortex and frontal operculum/Broca's area, superior temporal Sulcus/Wernicke's area and inferior temporal gyrus. Thus, participants who learned to meditate showed various changes in brain activity in prefrontal areas and an increase in activity in regions that underlie the bottom-up processing of emotional stimuli, suggesting that these participants recruited more brain areas involved in both evaluative processing and interoceptive awareness.

Hölzel et al. (2013) relied on whole-brain analysis to compare the effects of MBSR with a stress management control program for participants with generalized anxiety disorder. Participants were required to name the emotions of facial expressions (neutral, happy, or angry). A significant interaction between group and time was found for angry faces. In comparison to the control group, the meditation group showed greater activation of the right rostral middle frontal cortex and the right pars opercularis, reaching the insula (anterior insula), after intervention. There was no interaction or main effect for amygdala activity. Hence, MBSR helped participants recruit a prefrontal area that reached the insula, indicating the recruitment of top-down mechanisms when processing angry faces, even without explicit instructions to regulate responses. However, the amygdala activity was not significantly different, so this interpretation must be considered with caution.

In the same direction, another study (Goldin et al., 2012) investigated participants with social anxiety disorder through ROI analysis (the posterior cingulate cortex—PCC, dorsomedial prefrontal cortex—dmPFC, and vmPFC) as well as whole-brain analysis of BOLD signals. Participants in both groups (MBSR vs. aerobic exercises) were instructed to read adjectives of positive and negative social traits and reflect on whether the word applied to themselves (reactivity condition); results from this condition were compared to a neutral condition in which participants had to simply decide whether the letters of the adjective were upper case. There was a significant interaction between group and time. In particular, according to ROI analysis, meditators exhibited greater activity in the PCC when reacting to negative adjectives than that in the neutral condition, but there were no significant differences in dmPFC and vmPFC activity. Whole-brain analysis showed that, compared to the neutral condition, within the meditation group, there was increased activity in the vmPFC, left vlPFC, bilateral dorsolateral PFC (dlPFC), PCC/precuneus, left inferior parietal lobe (IPL), and left posterior superior temporal gyrus (STG) during the processing of negative adjectives (reactivity). Thus, people who took part in the meditation intervention showed increased activity in regions related to cognitive and attentional control during the processing of negative social traits, suggesting they were better able to use cognitive control during an emotional reactivity condition, even though they were not instructed to regulate emotion.

Another study from the same research team (Goldin et al., 2013) investigated the same sample as Goldin et al. (2012) but used a modified version of the emotional paradigm. Participants were exposed to negative beliefs about social anxiety (e.g., I am incompetent; others think I am not normal) and were instructed to react to them, reflecting on whether those beliefs applied to themselves (reactivity condition). There was a significant interaction between group and time in the reactivity condition compared to neutral condition (reading neutral statements). The group who underwent the MBSR intervention showed reduced activity in the right vlPFC when reacting to negative stimuli, whereas the control group exhibited increased activity in the same region. These results suggest that the participants who took part in the MBSR intervention reacted to negative stimuli with less cognitive regulation compared with a control group.

##### Emotion regulation

Among the experimental studies, only the study by Goldin et al. (2013) manipulated the paradigm to assess the regulation process itself, by instructing their participants to regulate their emotional responses with metacognitive attention to the present moment. This additional condition was compared with the reactivity response to the emotional stimuli (see description above). There was a significant interaction between group and time. During the regulation condition (compared to the reactivity condition), participants who underwent the MBSR training showed reduced activity in the right posterior STG and bilateral lingual gyrus and increased activity in the left IPL, right anterior IPL, right posterior IPL, and right superior parietal lobe (SPL). Thus, for those who practiced meditation for 8 weeks, the neural correlates of emotion regulation consisted of reduced activity in the posterior regions of the cortex and increased activity in the parietal regions, suggesting that attentional control played a role in the regulation of negative emotions for people with anxiety disorder.

#### Quasi-experimental studies

##### Emotional reactivity

The study by Froeliger et al. (2012) performed a whole-brain analysis of BOLD signals and compared the results obtained from Hatha Yoga practitioners with a control group. Participants were exposed to negative and neutral images during an emotional Stroop task (passive viewing condition). A significant interaction between group and valence was observed. In particular, the control group showed increased activity in the right dlPFC when viewing negative images, whereas the meditator group showed reduced activity in the same region when viewing both negative and neutral images. Hence, the control group used more top-down mechanisms to deal with negative emotion than long-term meditators.

Lutz et al. (2016) assessed the neural activity of Vipassana meditators in comparison to a control group through whole-brain analysis of BOLD signals. Both groups viewed negative, positive, and neutral adjectives related to personality (e.g., talents, social aspects, appearance), which were presented within four conditions: self-critical, negative non-self-critical, self-praising, and neutral. Compared to the control group, meditators showed increased activity in the dmPFC in the self-critical condition compare to the neutral condition. Hence, during exposure to aversive self-critical stimuli, long-term meditators recruited a brain region known to be associated with the cognitive aspect of emotional processing, suggesting that these participants were more conscious of emotions.

Lee et al. (2012) conducted whole-brain analysis of BOLD signals to compare Theravada meditators with beginners who meditated for 1 week (three times a day). Participants viewed happy, sad, and neutral images and were assessed during both a meditation and baseline states. There was a significant interaction between group and state. During the passive viewing of sad images (baseline), long-term practitioners exhibited greater activity of the left SFG relative to beginners. This finding indicates that long-term meditators regulated their emotions when exposed to negative emotional stimuli by relying on an attentional regulation region.

Laneri et al. (2017) compared Zen and Vipassana practitioners with a control group (non-meditators) through whole-brain analysis of BOLD signals and ROI analysis. The paradigm consisted of drawings of people in embarrassing scenes accompanied by sentences describing the situations. Participants were required to judge the level of embarrassment of the people being portrayed in these situations. There were no significant results for either the whole-brain and ROI analyses.

The study by Taylor et al. (2011) compared long-term Zen meditators with beginners that meditated for 1 week (20 min a day). Both groups viewed positive, negative and neutral images, but there were no significant interactions between group and valence in the reactivity condition.

##### Emotion regulation

Froeliger et al. (2012) investigated emotion regulation by analyzing a condition in which participants had to control the interference of negative and neutral images during an emotional Stroop task. There was a significant interaction between group and valence. During the interference control condition, meditators showed greater activation of the left vlPFC for negative images than for neutral ones, suggesting long-term meditators were better able to recruit cognitive resources that allowed them to disengage from the images and perform the attentional task.

Taylor et al. (2011) used ROI analysis to investigate the activity of the amygdala, insula, putamen, caudate nucleus, hippocampus, dorsal and rostral-ventral ACC, mPFC, lateral PFC, and orbitofrontal cortex (OFC) during a paradigm in which positive, negative and neutral images were presented to participants. To assess the regulation process, participants meditated for 2 min prior to image presentation so they could view images in a mindful state. There was a significant interaction between group and valence. Compared to long-term practitioners, beginners showed reduced activity in the left amygdala for negative images (subtracted from neutral images). There were no significant results for the other regions of interest. Hence, the mindful condition of beginners possibly downregulated their emotional responses to negative stimuli.

In the study by Lee et al. (2012), which compared Theravada meditators with beginners (1-week practice) using whole-brain analysis of BOLD signals, the regulation process also consisted of participants entering a meditative state before the paradigm. After 30 min of meditation, participants were exposed to happy, sad, and neutral images. The results showed an interaction between group and state, such that long-term practitioners showed greater activity in the left SFG when viewing sad images after the 30-min practice. Within the control group (beginners), there was an increase in the activity of the right inferior frontal gyrus (IFG) for sad images, relative to their baseline state (passive viewing). Hence, after inducing a meditative state, long-term practitioners and beginners appeared to rely on regions related to attentional control to modulate emotional processing.

Another study assessed the regulation process by dividing meditators (Zen and Vipassana) into two conditions in addition to a control group: those who meditated for 8 min before the paradigm and those who rested for the same period before the task (Laneri et al., 2017). Significant differences in the results from the whole-brain analysis were not observed. In the ROI analysis, the authors found that meditators who meditated before the paradigm showed reduced activity in the left anterior insula when exposed to negative stimuli compared to individuals who rested prior to the task. No differences were observed when comparing long-term meditators with the control group (non-meditators). Thus, only experienced meditators who meditated prior to the task exhibited reduced activity in the insula.

### Main findings

In summary, of the 11 studies included in the review, six relied on an experimental design and five relied on a quasi-experimental design. Samples were mostly healthy (8 studies), and the type of meditation varied, including MBSR, mindfulness practices, Zen, Vipassana, Theravada, and Hatha. The paradigms used to investigate emotion reactivity and regulation were diverse and included a variety of stimuli (images from IAPS, faces, video, adjectives and phrases with hypothetical situations). Regarding the neural results, this review did not identify a unique pattern of activity for the processing of negative stimuli in both experimental and quasi-experimental designs. However, some results obtained under reactivity conditions showed that meditation was associated with a greater recruitment of prefrontal and frontal regions, suggesting that these participants used top-down mechanisms when facing emotional stimuli, even without explicit instructions to modulate emotion (Farb et al., 2010; Allen et al., 2012; Goldin et al., 2012; Lee et al., 2012; Hölzel et al., 2013; Lutz et al., 2016). However, two studies reported the opposite result (Froeliger et al., 2012; Goldin et al., 2013). Regarding emotional areas, three studies observed an increase in insular activity (Farb et al., 2010; Allen et al., 2012; Hölzel et al., 2013). Finally, four studies did not detect any significant differences between meditators and controls in any of the regions under the reactivity condition (Taylor et al., 2011; Allen et al., 2012; Desbordes et al., 2012; Laneri et al., 2017). Only five studies, considering both experimental and quasi-experimental designs, explored the emotion regulation condition. Three studies observed an increase in the activity of regions related to cognitive and attentional processing (Froeliger et al., 2012; Lee et al., 2012; Goldin et al., 2013). One study observed reduced activity in the insula only for long-term meditators (Laneri et al., 2017), and one study observed reduced activity in the amygdala, but only for beginners (Taylor et al., 2011).

## Discussion

To our knowledge, this is the first study to systematically review evidence of how sitting and silent meditation modulates neural responses during the processing of negative visual emotional stimuli. It was not possible to determine a homogeneous pattern of brain activity related to the practice of meditation in participants. Nevertheless, some consistencies among study results should be highlighted. In general, there was a predominance of greater brain activity in prefrontal/frontal areas, suggesting increased recruitment of cognitive and attentional control resources in the processing of negative emotional stimuli due to meditation practices. Regarding emotion-related areas, there was a predominance of increased insular activity in meditators, suggesting that meditation might be associated with greater bodily awareness. Data will be discussed based on study design, i.e., experimental (naïve participants that underwent an intervention) and quasi-experimental (long-term meditators), and the type of outcome, i.e., emotional reactivity and emotion regulation.

### Experimental studies (naïve meditators)

#### Emotional reactivity results

All experimental studies used an intervention based on mindfulness, and most used paradigms that assessed reactivity, that is, participants were not instructed to regulate the processing of emotional stimuli but simply react to them. Notably, even without such instruction, the majority of studies demonstrated that participants who took part in a meditation training showed brain patterns that suggested the cognitive regulation of emotion. For example, in a sample of participants with social anxiety disorder (Goldin et al., 2012), there was increased activity in regions associated with cognitive and attentional control and emotion regulation (dmPFC, vmPFC, dlPFC, left IPL, and PCC) (Duncan and Owen, 2000; Miller and Cohen, 2001; Buhle et al., 2014; Kohn et al., 2014; Wang et al., 2017) while reacting to negative social traits. Interestingly, there was an association between increased activity in the dmPFC during the visualization of aversive stimuli (vs. neutral) and reduced functional impairments related to social anxiety disorder (Sheehan Disability Scale) and increased scores in mindfulness skills (Kentucky Inventory of Mindfulness Skills) (Goldin et al., 2012). These findings could suggest that the enhancement of mindfulness skills and the correlated prefrontal activity may mediate the effect of meditation on the reduction of social anxiety symptoms.

Increased recruitment of regions related to top-down emotion regulation was also observed in the studies by Farb et al. (2010), Allen et al. (2012), and Hölzel et al. (2013). Despite similar patterns, the modulated regions were not identical among these studies. In the study by Allen et al. (2012), only individuals with higher amounts of mindfulness practice (hours) showed increased activity in the mPFC and dorsal ACC, a region related to the cognitive control of emotion (Etkin et al., 2011). With respect to behavioral data, the amount of practice within the experimental group predicted improved stop accuracy in the Error Awareness Task, which indicated that participants were better able to withhold their responses in no-go trials during the task. Moreover, the experimental group was faster when performing in the incongruent condition of a Stroop task, suggesting better attentional control in the presence of aversive stimuli. The study by Hölzel et al. (2013) demonstrated that the experimental group showed increased activity in the right rostral middle frontal cortex and vlPFC (right pars opercularis). Interestingly, these participants showed a negative correlation between activity in the vlPFC and anxiety scores, as indexed by the Beck Anxiety Inventory (BAI). Thus, this study suggests that increased activity in the vlPFC played a role in the regulation of anxiety severity among those reporting a condition of generalized anxiety disorder. Farb et al. (2010) also demonstrated greater activation in areas related to cognitive control in the experimental group (vmPFC, right vlPFC, right SFG). However, this study also showed a deactivation in the left lateral PFC. As this study did not conduct a pretest assessment, these findings may have been influenced by the lack of a baseline condition. Moreover, the participants who took part in the meditation intervention showed significant training-related improvements in anxiety and depression scores (BAI and Beck Depression Inventory Second-Edition—BDI-II). Hence, these findings partially support the hypothesis that meditation practice activates regions related to cognitive control during emotional processing, in addition to improving anxiety and depression symptoms.

Only the study by Goldin et al. (2013) showed decreased prefrontal activity in participants who underwent meditation training, specifically, reduced activity in the vlPFC during the visualization of aversive stimuli (negative beliefs about social behavior) in comparison with a control group (aerobic exercise training). Previous evidence shows that increased activity in vlPFC is associated with negative emotion regulation (Buhle et al., 2014; Kohn et al., 2014). This result contrasts the findings of the studies discussed above, since there was a reduction in the activity of a region related to emotion regulation after meditation training in comparison with a control group who completed aerobic exercise training. The diversity of the negative emotional stimuli used, the studied populations and the control groups used are possibly factors underlying the controversies found among these studies. In Goldin et al. (2013) specifically, the population had anxiety, the stimuli were related to this anxiety condition and were thus highly negative. In addition, in this study the control group practiced aerobic exercise. One possibility is that the aerobic training increased prefrontal activity more than the meditation training over an 8-week intervention. In fact, evidence of an increase in activation in prefrontal and parietal cortexes, regions involved in attentional control, due to aerobic training has been suggested (Colcombe et al., 2004). Collectively, the majority of these studies suggest that people who participated in a meditation intervention are better able to recruit brain regions that underlie the top-down mechanisms of emotion regulation even without explicit instructions to regulate the presented aversive stimuli.

Importantly, three studies (Farb et al., 2010; Allen et al., 2012; Hölzel et al., 2013) demonstrated increased activity in the insula. This region has been consistently related to interoceptive awareness of internal and visceral states of the body as well as emotional and metacognitive self-consciousness (Craig, 2004; Fleming and Dolan, 2012). A recent systematic review showed that greater insular activation in participants after MBIs is likely related to enhanced present-moment awareness, a core feature of mindfulness meditation (Young et al., 2017). Together, these results might suggest that meditation helps enhance regulation in two ways: through improved top-down control and through improved awareness of bodily and emotional states. These findings are in line with the notion that meditation helps foster the subjective experience of the acceptance of internal experiences and states (Grant et al., 2011). Such an assumption is consistent with the hypothesis that meditation helps promote a particular type of emotion regulation strategy (Menezes et al., 2012; Opialla et al., 2015; Guendelman et al., 2017) in which both attentional control and an awareness of bodily and emotional states subserve more adaptive emotion regulation.

One region that is widely known to be involved in emotional reactivity is the amygdala. This region is also involved in the interactions between emotions and attention, as it has been repeatedly implicated in the emotional processing of negative and positive stimuli and the facilitation of attentional orientation to the emotional relevance of these stimuli (Vuilleumier, 2005; Phelps, 2006; Pessoa, 2008). In the study by Desbordes et al. (2012), participants who participated in the meditation intervention showed reduced activity in the right amygdala when considering positive, negative, and neutral conditions together, with a trend toward reduced activity under the negative condition. Although this result is consistent with the published findings, it should be considered with caution as it is based in a trend toward decreased amygdala activity. Anxious individuals who learned meditation showed reduced amygdalar activity when instructed to regulate the processing of negative emotional stimuli (Goldin and Gross, 2010). Reductions in amygdalar activity have also been reported in studies assessing the use of cognitive regulatory strategies (Ochsner et al., 2002, 2004; Mocaiber et al., 2011; Sanchez et al., 2015). In fact, the literature on emotion regulation strategies suggests that one of the most investigated regulatory strategies—cognitive reappraisal—is often accompanied by a neural pattern of increased activity in the frontal/prefrontal regions and decreased activity in limbic regions (Gross, 1998, 2002; Buhle et al., 2014; Etkin et al., 2015).

#### Emotion regulation results

Only one experimental study explicitly required participants to regulate emotional responses by requiring them to use metacognitive attention to the present moment and demonstrated that participants who learned meditation were better able to recruit parietal regions, suggesting that they relied on the attentional regulation of emotions (Goldin et al., 2013). This finding was corroborated by a decrease in the subjective experience of negative emotions, which was associated with amount of practice within the meditation group. Greater amount of practice were also associated with increased activity in the left IPL and mPFC. The finding that naïve meditators relied on parietal and prefrontal regions to regulate negative emotions is consistent with previous data demonstrating that these regions underlie attentional and cognitive strategies that enable the modulation of the negative impact of emotional cues (Mocaiber et al., 2011; Ferri et al., 2013; Buhle et al., 2014; Kohn et al., 2014; Wang et al., 2017). Thus, one way that meditation may help regulate emotion is through top-down mechanisms.

### Quasi-experimental (long-term meditators)

#### Emotional reactivity results

Two studies found that long-term meditators showed increased activity in the dmPFC (Lee et al., 2012; Lutz et al., 2016). This region is involved in the cognitive processing of emotions and its activation is related to the conscious evaluation of threats (Mechias et al., 2010; Etkin et al., 2011). Indeed, in the study by Lutz et al. (2016), participants were instructed to pay attention to the meaning of negative adjectives and the internal responses triggered by these words, which might have required greater evaluative processing. This result is in line with the finding that being mindful of emotions and the body, compared to cognitively reflecting about oneself, leads to greater activation in the dmPFC in healthy subjects (Scherpiet et al., 2015). In addition, Modinos et al. (2010) found that trait mindfulness was correlated positively with dmPFC activation in a condition in which participants had to reappraise negative images. Greater activity in another frontal area (left SFG) in long-term practitioners relative to beginners (1-week practice) during the visualization of negative stimuli was demonstrated by Lee et al. (2012). In addition, long-term meditators showed significantly fewer omission errors in a cognitive task compared to beginners and showed significantly higher scores in mindfulness skills, as measured by the Toronto Mindfulness Scale and the Cognitive and Affective Mindfulness Scale-Revised. Thus, these findings suggest that long-term meditators have better attentional control, which likely subserves the improved regulation of negative emotions and performance on a cognitive task (Lee et al., 2012).

One study found reduced activity in a prefrontal region related to emotion regulation. In particular, long-term practitioners reduced activity in the right dlPFC during the visualization of negative and neutral stimuli (Froeliger et al., 2012) as compared to non-meditators. This finding contradicts the data reported in the studies above, since there was reduced activity in a prefrontal region related to emotion regulation. Importantly, two aspects must be considered when trying to understand this contradiction. First, the emotional paradigm intercalated reactivity and emotion regulation conditions in a randomized way, which may have caused difficulty in switching between conditions, specifically for non-meditators. The emotion regulation condition was a high-load attentional task that demanded intense cognitive control. Perhaps non-meditators had less cognitive flexibility and sustained high prefrontal activity throughout the session. Accordingly, the apparent reduction in prefrontal activity in meditators compared to the control group may be due to a failure of the control group to adjust prefrontal recruitment in the low attentional demand condition of passive viewing emotional stimuli (the emotional reactivity condition). Second, the sample for this study was considerably small, with only 7 participants in each group.

#### Emotion regulation results

A pattern of increased activity in frontal and prefrontal regions was observed within the quasi-experimental studies that investigated the emotion regulation process *per se*. For instance, long-term meditators showed greater activation of the vlPFC when performing an attentional task in which they had to modulate the interference of negative stimuli (Froeliger et al., 2012). Notably, during the reactivity condition (passive viewing of the images), these meditators did not recruit prefrontal regions to process the emotional stimuli. Hence, when performing in a regulation condition, meditators were better able to engage in the attentional demands of the task and more efficiently recruit the vlPFC, a region known to indicate cognitive control. Thus, the activity of the vlPFC might have helped in the control of emotion interference among long-term meditators (Froeliger et al., 2012). This suggestion is in line with the finding that during the cognitive reappraisal of aversive images, greater activity in this region was correlated with a reduction in the subjective experience of negative emotions (Wager et al., 2008). However, it should be noted that in the study by Froeliger et al. (2012), the behavioral results were not significantly different between groups in terms of either accuracy or reaction time, so inferences about emotion regulation are only based on neural responses.

Lee et al. (2012) demonstrated that both long-term practitioners and beginners (1-week practice) showed increased activity in frontal regions during the regulation of negative stimuli, wherein they viewed images in a meditative state. The right IFG was more highly activated in beginners. This region has been associated with inhibitory control (Rubia et al., 2003), and, more recently, Hampshire et al. (2010) demonstrated that the recruitment of this region was related to the detection of important cues, regardless of whether these cues were associated with inhibitory motor responses. As participants had to categorize the valence of the emotional images in the study by Lee et al. (2012), perhaps the detection of sad images lead to greater activity in the right IFG. In the long-term meditators, there was greater activity in the left SFG in both the reactivity (passive viewing) and regulation (meditative state) conditions when compared to the beginners in the reactivity condition. The activation of this region was also observed in a study in which expert meditators performed an attentional task (Brefczynski-Lewis et al., 2007). Hence, meditation appears to foster the more efficient use of attentional resources, which facilitates reduced emotional reactivity and greater emotional stability (Lutz et al., 2008).

Regarding the neural structures related to the bottom-up processing of emotional stimuli, Taylor et al. (2011) found that only beginners (1-week practice) but not long-term practitioners showed reduced activity in the left amygdala when attempting to regulate (mindful state) negative emotions. Goldin and Gross (2010) and Leung et al. (2017) also observed that after a meditation intervention, individuals showed reduced amygdala activity during the processing of negative emotional stimuli. Hence, beginners were able to reduce the processing of negative stimuli by decreasing amygdala activity. Interestingly, both beginners and long-term meditators reported a reduction in the subjective experience of emotion intensity. Therefore, one hypothesis is that more experienced meditators might not need to modulate the bottom-up processing of emotion, as indexed by amygdala activity, to not feel impacted by it, whereas beginners rely on the attenuation of the bottom-up mechanisms.

Laneri et al. (2017), on the other hand, did not observe significant differences between long-term meditators and non-meditators. Significant differences were only detected when the two subgroups of meditators (the group that meditated prior to the task and the group that did not meditate) were compared. Meditators who meditated showed reduced activity in the left anterior insula toward negative stimuli. The recruitment of the insula has been systematically observed among meditators and interpreted as an index of enhanced metacognition and bodily consciousness (Fox et al., 2016; Gotink et al., 2016). Therefore, in the study by Laneri et al. (2017), the reduced activity of the insula may suggest that the long-term meditators who meditated prior to the task might have relied less strongly on the interoceptive awareness of the social pain of others. Notably, this study was the only one to assess social pain as an aversive stimulus. Hence, discrepancies concerning insular activity might have resulted from differences in experimental paradigms.

### Limitations

A limitation of this systematic review is the large between-studies differences in designs, analyses and the types of paradigms used to investigate emotional reactivity and emotion regulation. Furthermore, the small number of studies included in this review precluded us from performing a coordinate-based meta-analysis, considering the guidelines for the minimum number of experiments to satisfactorily conduct these quantitative analysis (Eickhoff et al., 2016). Thus, as the review is qualitative, we were not able to easily assess whether the regions are exactly the same across studies. Moreover, sample sizes were generally small, many studies did not use an active control group, and no studies performed follow-up assessments. Among the quasi-experimental studies, various types of meditation practices and lengths of previous experience were reported. These disparities, as well as the limited details provided in the publications about the dose and duration of training and characteristics of each type of practice, precluded us from drawing conclusions about factors that might interfere with the results. Although the use of a control group was an inclusion criterion, some results reported in this review were obtained from within group comparisons (e.g., meditators group: pre vs. post intervention). Importantly, the exact contrast condition for each result is clearly described in the results section and in Tables 1, **2**. Finally, we did not include unpublished studies and did not assess the quality of the studies or effect sizes; therefore, the conclusions may be affected by possible biases.

## Final considerations

The present systematic review revealed that a homogeneous pattern of brain activity related to the practice of sitting and silent meditation is not detected in participants exposed to negative visual stimuli. Nevertheless, one consistent result among the studies was the increased prefrontal/frontal activity in meditators, which might indicate the increased recruitment of cognitive and attentional control resources in long-term meditation practitioners and in participants who participated in a meditation intervention. This increased frontal activity was also observed under emotional reactivity conditions, where participants were asked to simply react to negative stimuli, suggesting that meditation practice prompts regulatory mechanisms when participants face aversive stimuli, even without an explicit request. Notably, modifications of activity in some regions (dlPFC, medial PFC, anterior cingulate cortex, and insula) have also been reported in studies investigating meditation practices, regardless of the emotional context (Hölzel et al., 2007; Baron Short et al., 2010; Fox et al., 2016). With respect to emotion-related areas, among the studies that reported any modulation induced by meditation practice, most of results noted increased insular activity in meditators. This result is consistent with the hypothesis that meditation helps foster an interoceptive awareness of bodily and emotional states. Finally, when considering the amount of meditation practice, an increase in the amount of practice improves neural and behavioral mechanisms that address negative emotions. In summary, despite the methodological limitations noted above, the reviewed studies provide insights into the potential clinical use of meditation to foster healthier emotion regulation processes.

## Author contributions

AM collected the data, developed and tested the search strategy, contributed to data analysis, and manuscript preparation. LO developed the search strategy, contributed to data analysis, and manuscript preparation. MP developed the search strategy, contributed to data analysis, and manuscript preparation. CM developed the search strategy, contributed to data analysis, and manuscript preparation.

### Conflict of interest statement

The authors declare that the research was conducted in the absence of any commercial or financial relationships that could be construed as a potential conflict of interest.
